# Efficacy of the GLP-1 receptor agonist, semaglutide, in abstinence from illicit and nonprescribed opioids in an outpatient population with treatment-refractory OUD: A randomized, double-blind, placebo-controlled clinical trial protocol

**DOI:** 10.21203/rs.3.rs-6666196/v1

**Published:** 2025-05-26

**Authors:** Christopher S. Freet, Kirsten Shuler, Sarah Kawasaki, Eric Weintraub, Aaron Greenblatt, Mat Kladney, Edward Nunes, Katrina L. Foster, Lan Kong, Nazia Raja-Khan, H. Harrington Cleveland, Patricia S. Grigson, Scott C. Bunce, Timothy R. Brick, Jennifer E. Nyland

**Affiliations:** Penn State College of Medicine; Penn State College of Medicine; Penn State College of Medicine; University of Maryland School of Medicine; University of Maryland School of Medicine; New York University Grossman School of Medicine; Columbia University Irving Medical Center; National Institute on Drug Abuse; Penn State College of Medicine; Penn State College of Medicine; Pennsylvania State University; Penn State College of Medicine; Penn State College of Medicine; Pennsylvania State University; Penn State College of Medicine

**Keywords:** glucagon-like peptide 1 receptor agonist, GLP-1RA, craving, substance use disorder (SUD), semaglutide, ecological momentary assessment (EMA), opioid use disorder (OUD)

## Abstract

**Background:**

Standard medications for opioid used disorder (MOUD) provide effective treatment pathways for recovery compared with no treatment or behavioral therapies alone. That said, treatment-refractory opioid used disorder (OUD) often can limit effectiveness and contribute to high attrition and relapse rates. Novel, more effective approaches are needed for the treatment of OUD. To that end, glucagon-like peptide 1 receptor agonists (GLP-1RAs) provide a promising option as a non-opioid pharmacological intervention for OUD. Current data suggest that GLP-1RAs decrease craving measures in a residential OUD population but, to date, no controlled clinical trials have been conducted to determine if a GLP-1RA can increase abstinence for substance use and reduce craving in individuals with OUD in an outpatient population. The purpose of the current protocol is to evaluate the potential for the GLP-1RA, semaglutide, to effectively increase abstinence and reduce craving in an outpatient population enrolled in a MOUD program and experiencing treatment refractory OUD.

**Method:**

This protocol is a randomized, double-blind, placebo-controlled clinical trial designed to test the efficacy of the GLP-1RA, semaglutide, in 200 participants enrolled in an outpatient MOUD program (n = 100 buprenorphine; n = 100 methadone) for the treatment of OUD. Outcomes include the probability of participants being abstinent from illicit and nonprescribed opioids, as well as measures of craving and days of drug use. Measures will be evaluated using urine toxicology screens and self-report assessments across 19 weeks during a screening visit (Study Week 1), 12 treatment visits (Study Weeks 2–13), a washout visit (Study Week 14), and a final follow-up visit (Study Week 19).

**Discussion:**

This manuscript describes a phase II clinical protocol to collect data on the efficacy of a GLP-1RA, semaglutide, in persons enrolled in a MOUD program and experiencing treatment-refractory OUD. Completion of the current project will support the feasibility of phase III clinical trials for further evaluation in larger outpatient OUD populations that may lead to a new indication for GLP-1RA as a novel and effective treatment for OUD.

**Trial registration:**

ClinicalTrials.gov: NCT06548490. Registered 12 August 2024, https://clinicaltrials.gov/study/NCT06548490

## Background

Pharmacological treatments for opioid use disorder (OUD) continue to be limited to three medications in the United States: buprenorphine (BUP), methadone, and naltrexone. While these standard medications for opioid use disorder (MOUD) offer effective pathways for recovery compared to no treatment or behavioral therapies alone (1, 2), patients treated with MOUDs still experience high rates of attrition and relapse (3, 4). For cases of treatment-refractory OUD, where patients continue to use drugs despite the use of these approved MOUDs, the consequences can include adverse health and social consequences, including a heightened risk of overdose. In these cases, few treatment options remain. Although slow-release oral morphine (SROM) and Injectable Opioid Agonist Treatment (iOAT) are available in select Canadian provinces and in several countries in Europe (5–7), these treatment options are prohibited by the Controlled Substances Act (8) in the United States. Consequently, there is a continued need for effective, non-opioid-based treatments to utilize as an alternative to, or adjunct with, standard MOUD therapies.

A current and promising avenue of interest is the use of glucagon-like peptide-1 receptor agonist (GLP-1RA) medications to treat OUD. These medications aim to address the craving inherent in OUD and to increase the efficacy of treatment in cases involving refractory OUD. Peripherally, GLP-1 is a hormone produced by enteroendocrine L cells in the small intestine that reduces food intake, in part, by increasing insulin release, decreasing glucagon release, and decreasing gastric emptying (9). Centrally, GLP-1 is an endogenous neuropeptide produced in the caudal portion of the nucleus of the solitary tract (NST). Efferent projections from the NST terminate in a wide range of dorsomedial and paraventricular hypothalamic nuclei and in mesolimbic nuclei such as the ventral tegmental area and the nucleus accumbens (10, 11), allowing GLP-1 to play a central role in the regulation of feeding behavior (12, 13). Currently, GLP-1RAs are approved by the Food and Drug Administration (FDA) and utilized in the treatment of Type 2 diabetes mellitus, as well as in obesity, in humans.

There is a growing body of literature, however, demonstrating that GLP-1RAs also decrease intake and motivation for a number of addictive substances, suggesting that these agonists may be an effective treatment for substance use disorder (SUD). Published clinical trials, to date, have investigated the GLP-1RAs, exenatide, dulaglutide, and semaglutide in persons with SUDs involving alcohol, nicotine, and cocaine. For example, a double-blind, placebo-controlled clinical trial using exenatide in patients with alcohol dependence used functional magnetic resonance imaging (fMRI) to demonstrate attenuated cue reactivity to alcohol in the ventral striatum and septal area. However, heavy drinking days and total alcohol intake were not significantly different compared with placebo, with the exception of a subgroup with class I obesity or greater (14, 15). Another double-blind, placebo-controlled, randomized clinical trial used semaglutide and found a reduction in alcohol consumed in a laboratory self-administration task, a reduction in the number of drinks/drinking day, and a reduction in alcohol craving (16). A double-blind, placebo-controlled, randomized clinical trial demonstrated that exenatide, in combination with nicotine replacement therapy, attenuated craving and withdrawal and increased smoking abstinence in prediabetic and/or overweight treatment-seeking smokers (17, 18). Whereas a daily treatment study found semaglutide was associated with a reduction in the number of cigarettes smoked per day (16), another study evaluating the efficacy of dulaglutide plus varenicline in a smaller subgroup was found to be ineffective for smoking cessation (19). Finally, with respect to cocaine, a double-blind, within-subject design study using acute exenatide pre-treatment in patients with cocaine use disorder did not observe a change in cocaine intake, wanting, or self-reported euphoria. Using only one dose of exenatide with a single acute treatment may have limited the ability to draw clear conclusions regarding efficacy from this study (20). According to ClinicalTrials.gov, several clinical trials are underway (NCT05895643, NCT05891587, NCT05892432, NCT06015893, NCT05895643) that evaluate the effects of semaglutide on alcohol consumption and/or cue-elicited craving in alcohol use disorder (AUD). Additionally, two recently completed clinical trials (results pending) evaluated the effect of the GLP-1RA liraglutide (NCT03712098) and semaglutide (NCT05530577) on nicotine intake and smoking cessation.

The ability of GLP-1RAs to modulate intake and motivation appears to extend to opioids as well, with current preclinical and clinical literature supporting such a hypothesis. For example, in preclinical studies, GLP-1RAs decrease drug self-administration (21–23) and drug-seeking/reinstatement behavior (24–27) for heroin, oxycodone, and fentanyl in rats. Clinically, we recently conducted a pilot randomized, double-blind, placebo-controlled clinical trial evaluating the efficacy of the GLP-1RA, liraglutide, on the reduction of craving in a residential population receiving treatment for OUD (28, manuscript in prep). Although this study was small and preliminary, we observed a 40% reduction in ambient craving, as measured by ecological momentary assessment (EMA), in the liraglutide group compared with the placebo control. This effect was consistent across all tested doses of liraglutide (0.6 mg, 1.2 mg, and 1.8 mg). In addition to the clinical trial proposed in the current protocol, which targets a treatment-refractory population, there is also a double-blind, placebo-controlled, randomized trial (NCT06639464) set to begin recruitment to determine the effects of semaglutide on cue-reactivity among individuals who are newly initiating BUP treatment for OUD.

### The current study

Given the increased focus on the use of GLP-1RAs to treat OUD, and to further investigate our previous GLP-1RA findings suggesting reduced craving, we propose the current protocol to evaluate whether the GLP-1RA, semaglutide, will increase abstinence from opioid use in individuals in an outpatient treatment setting for OUD, specifically in those who continue to use opioids after initiating BUP or methadone treatment. The described study is a randomized, double-blind, placebo-controlled, parallel-arm study evaluating once-weekly treatment with semaglutide compared with placebo. Opioid use will be assessed using urine toxicology screens and self-report assessments. Smartphone surveys and in-person craving scales will be used to obtain measures of cravings across 19 weeks during a screening visit, 12 treatment visits, a washout visit, and a final follow-up visit.

The current protocol will employ two methods to detect illicit substance use: self-report assessments, specifically the Timeline Followback (TLFB) method, and biological testing methods using urine samples. Participants will be informed that the urine testing and self-report assessments will take place in a clinical trial setting where there are no consequences associated with substance use. While the effectiveness and utility of these methods can vary based on the type of drug, sample type, and testing environment, a 2023 systematic review and meta-analysis (29) reported that overall agreement between biological testing methods and self-report ranged from good to excellent. In clinical trials where there are no consequences, self-reports tend to be more reliable and agreement between self-report and urine testing is higher when participants are aware that biological testing will occur (29).

Craving, a multidimensional phenomenon with physiological, neurochemical, subjective, and behavioral correlates (30), has been central to theories of addiction for more than seven decades, particularly as a predictor of continued use and relapse to substance use, including opioids (31, 32). We will evaluate the subjective aspect of craving in the current study using EMA before, during, and following the study treatment. EMA assessments of craving during residential OUD treatment and daily diary approaches to measure indicators of recovery wellbeing and risk in recovery community centers (RCCs) have been shown to have high reliability, sensitivity, and utility, suggesting that these methods are effective ways to measure craving and recovery-related metrics in these populations (33–35).

## Methods

### Aim

The purpose of this study is to determine whether 12 weeks of once-weekly treatment with the GLP-1RA, semaglutide, will reduce illicit opioid use over a 19-week period (133 days) among individuals in outpatient treatment for OUD, and who are still experiencing positive urine screens despite receiving either BUP for at least 14 days or methadone maintenance treatment for at least 28 days (i.e., MOUD).

### Endpoints

The primary endpoint for this study is the **1) Probability of participants being abstinent from illicit and nonprescribed opioids** (Time Frame: weekly for 12 weeks; study weeks 2 through 13). Each week in the 12-week trial period will be rated as abstinent if both urine test and participants’ report by TLFB are negative for illicit/non-prescribed opioids, or urine is negative and TLFB missing, or TLFB negative and urine missing; and not abstinent otherwise (either urine positive, or TLFB positive, or both are missing). For fentanyl, declining concentration in urine will be considered negative for usage.

The secondary endpoints for this study are: **1) Self-reported opioid craving scores as assessed via smartphone surveys as compared to control group, controlling for baseline** (Time Frame: daily in bursts over 12 weeks). **2) Self-reported opioid craving as assessed via In-person Cravings Scales scores collected from participants at weekly visits as compared to control group, controlling for baseline** (Time Frame: weekly for 12 weeks; study weeks 2 through 13). **3) Binary indicator of sustained abstinence from opioids–weekly abstinence rating (see primary outcome above) is rated as abstinent over the last 4 weeks of the treatment period** (Time Frame: weekly for 4 weeks; study weeks 10, 11, 12, and 13). **4) Binary indicator of abstinence from stimulants (i.e., cocaine or non-prescribed amphetamines) (urine negative, TLFB negative) over the last 4 weeks of the treatment period** (Time Frame: weekly for 4 weeks; study weeks 10, 11, 12, and 13). **5) Days using opioids over the 12-week treatment period by TLFB** (Time Frame: weekly for 12 weeks; study weeks 2 through 13). **6) Days using stimulants over the 12-week treatment period by TLFB** (Time Frame: weekly for 12 weeks; study weeks 2 through 13). **7) Association over the 12-week treatment period between smartphone survey measures of craving and abstinence from opioids and other drugs, as indicated by negative urine drug screens** (Time Frame: daily in bursts over 12 weeks; study weeks 2 through 13).

### Study Setting and Recruitment

Participants will be recruited across three sites: 1) the Pennsylvania Psychiatric Institute (PPI), Harrisburg, PA, USA; 2) the University of Maryland Baltimore (UMD), Baltimore, MD, USA; and 3) the New York University (NYU) at the Bellevue Hospital Center, New York, NY, USA.

Electronic medical records (EMRs) for patients receiving outpatient treatment for an OUD at each of the study sites will be reviewed against the study inclusion/exclusion criteria by a member of the study team at that site (pre-screening). The study teams at each site will also contact other treating physicians at clinics providing medication treatment for OUD, alerting them to the availability of the study and the criteria for eligibility and encouraging them to refer potential participants to the study. Potential participants will be those identified as having an established diagnosis of OUD and who appear likely to meet the inclusion, and not the exclusion criteria. Once pre-screening has been completed, potentially eligible patients will be approached at their clinic visit where the physician/staff will briefly explain the proposed study and ask the patient if they would be interested in discussing further with a study member. If the patient is interested, a trained, IRB-approved study team member will discuss the full study with them. Potential participants will be recruited at the time of a regularly scheduled clinical visit and prior to any study procedures taking place.

The study was approved by the Pennsylvania State University Institutional Review Board (IRB). An IRB-approved study team member will meet with the potential participant in a quiet private area. After a careful and complete explanation of the study details, risks, and other options are provided to the potential participant, a copy of the consent form will be provided to them for review. If they are interested in consenting to the study, they will be given additional time to ask questions. When all questions have been answered to their satisfaction by a study team member, the potential participant will be asked to sign the informed consent document. Study team members will stress that participation is completely voluntary. Additionally, it will be carefully and clearly explained that if the potential participant decides not to participate in the study, that decision will have no impact on the clinical care they will receive. Similarly, study enrollment will not interfere with or delay the administration of standard OUD therapies, e.g., behavioral treatments and/or MOUD.

### Inclusion/Exclusion Criteria

Inclusion criteria include: 1) Age 18 to 75 years; 2) Body mass index (BMI) ≥ 18; 3) Able and willing to provide informed consent prior to any study-related activities; 4) Current diagnosis of Diagnostic and Statistical Manual of Mental Disorders (DSM)-5 OUD as per the Mini International Neuropsychiatric Interview (MINI) or per the site clinic diagnosis. Patients are eligible if they have a MINI > 3 (“moderate” or “severe” in the “Specify If” box in the Substance Use Disorder (Non-Alcohol) module for the category of opiates); 5) Currently receiving outpatient treatment for OUD and at least 2 weeks on BUP or 4 weeks on methadone at the study site and/or at an associated clinic; 6) Have at least 1 urine test positive for opioids after 2 weeks on BUP or 4 weeks on methadone; 7) Have positive self-reporting of opioid use after 2 weeks on BUP or 4 weeks on methadone; 8) If anatomically capable of becoming pregnant and of childbearing age, is not pregnant (confirmed) or breastfeeding at the time of enrollment and agrees to use a medically accepted method of birth control or to abstain from sexual intercourse while in the study; 9) Able to read and communicate in English to the level required to accept standard care and complete all study requirements; 10) Able and willing to engage/adhere to the entirety of the study protocol (19 weeks); 11) Not currently a prisoner.

Exclusion criteria include: 1) Age < 18 or > 75 years; 2) BMI < 18; 3) Individuals who are pregnant, planning pregnancy, breastfeeding, or unwilling to use adequate contraceptive measures at the time of enrollment; 4) Current use of GLP-1R agonist; 5) History of angioedema, serious hypersensitivity reaction, or anaphylactic reaction to semaglutide or another GLP-1R agonist; 6) Personal or family history of medullary thyroid carcinoma (MTC) or patients with multiple endocrine neoplasia syndrome Type 2 (MEN 2) or thyroid nodule; 7) Type 1 diabetes or history of diabetic ketoacidosis; 8) Type 2 diabetes mellitus or current use of a dipeptidyl peptidase-4 (DPP-4) inhibitor; 9) Past 30-day use of Sincalide, sulfonylureas, insulin and insulin products or other medications that may interact with semaglutide; 10) Hypoglycemia on intake visit (blood glucose < 60 mg/dL); 11) End-stage renal failure, on dialysis, or glomerular filtration rate (GFR) < 30mL/min per 1.73 square meters or previous renal transplant; 12) End stage liver disease or previous liver transplant; 13) Current or past diagnosis of pancreatitis, gastroparesis, or other severe gastrointestinal (GI) disease; 14) Current or past diagnosis of gallbladder disease or gallstones; 15) Serious cardiovascular disease within the past 6 months (e.g., uncontrolled hypertension, heart failure, significant cardiac arrhythmias, myocardial infarction, presence of angina pectoris, symptomatic coronary artery disease, deep vein thrombosis, pulmonary embolism, second- or third-degree heart block, mitral valve or aortic stenosis, hypertrophic cardiomyopathy, stroke); 16) Severe co-occurring psychiatric disorder (e.g., bipolar disorder, psychotic disorder, schizophrenia), and/or history or evidence of organic brain disease or dementia that would compromise safety or compliance with the study protocol in the opinion of the site principal investigator (PI) and/or physician; 17) Significant risk of suicide requiring a different/higher level of care, according to the clinical judgment of the study physician or site principal investigator, or history of suicide attempts within the past 1 year, unless participation is cleared by clinician assessment and/or judgment. A C-SSRS indicating a history of suicide attempts within the past year, or active suicidal ideation within the past 1 month, will qualify as significant risk of suicide; 18) Treatment with any investigational drug in the one month preceding the study; 19) Any contraindication to methadone, BUP, or a GLP-1R agonist; 20) Previous randomization for participation in this trial; 21) Any other condition at screening that precludes safe participation in the trial in the judgment of the site PI or study physician; 22) Plans for travel outside of the local area over the 19 weeks (1 week of baseline, 12 weeks of medication, 1 week wash-out, and follow-up after a further 28 days) that would interfere with visits during the study period or other logistic factors that would make it difficult to commit to the entire duration of study; 23) Currently a prisoner.

### Trial Design/Procedure

#### Intervention Allocation and Blinding

Semaglutide (Ozempic^®^, Novo Nordisk) is a GLP-1R agonist approved by the FDA as an adjunct to diet and exercise to improve glycemic control in adults with Type 2 diabetes mellitus, and to reduce the risk of major adverse cardiovascular events (cardiovascular death, non-fatal myocardial infarction, or non-fatal stroke) in adults with Type 2 diabetes mellitus and established cardiovascular disease. Semaglutide will be purchased through a central pharmacy in concentrations of 0.68 mg/ml and 1.34 mg/ml provided as a solution in pre-filled, multi-dose pens. The planned dosing schedule will start with the initial dose at 0.25 mg once weekly for 4 weeks. The dosage will then be increased to 0.50 mg once weekly for 4 weeks, and, finally, it will be increased to 1.0 mg once weekly for 4 weeks. Based on participant tolerability (for example, GI distress), the clinician can use their judgment, in consultation with the participant, to adjust the dose. Placebo will be a dry needle stick using an injector pen that mimics the clicking noise present in the semaglutide pen; no substances will be injected for placebo controls. Active drug or placebo will be administered via a subcutaneous injection in the abdomen.

The study medication will be blinded to the study teams and an unblinded clinical staff member/injector will be used to administer. Participants will be blinded using a draping of their abdomen area so that they cannot see the type of injection (semaglutide or placebo pen) being administered subcutaneously. Additionally, a cover sleeve will be placed on the semaglutide and placebo pens to ensure the participant remains blinded. Unblinded personnel at the site pharmacy will be available to handle the lists that reveal if the participant is receiving semaglutide or placebo.

#### Randomization

After the screening visit (Study Week 1), but before the first treatment visit (Study Week 2), participants will be randomized to either active study drug or placebo conditions. Randomization will be performed using the blockrand package (36) for the R statistical software. A randomization list stratified by site and MOUD, will be created using variable-size, random permuted blocks to ensure that the number of participants in each arm is balanced after each set of B randomized participants, where B is the block size. Block sizes are selected randomly to ensure full blinding. The Wear-IT server will automatically assign participants to each group following the randomization list and inform the appropriate distributing pharmacy; the pharmacy will then provide the blinded, randomized labelling prior to shipping the study drugs to the study site.

In the case of a medical emergency, when knowledge of the participant’s treatment assignment might influence the participant’s clinical care, the treating physician will be able to contact the site pharmacy to access the participant’s treatment assignment as per local site practices. Research team members will document the reasons for unblinding in the participant’s source documentation. Study team members will not share information with personnel involved with the analysis and conduct of the study.

#### Participant Timeline

Table 1 outlines the schedule of the study protocol. At the screening visit (Study Week 1), the PI will review the inclusion/exclusion criteria with the potential participant prior to obtaining informed consent to determine if they are initially eligible to participate in the trial. If the potential participant meets inclusion criteria and none of the exclusion criteria, informed consent will then be obtained prior to any research-only procedures. Following informed consent, participants will be asked to provide a urine sample that will be tested for the presence of opioids (including fentanyl), cocaine and other stimulants, benzodiazepines, barbiturates, cannabis, and MOUDs (methadone or BUP). If the urine sample is not positive for opioids, the participant will not be eligible to continue, and the screening visit will be terminated. If the urine sample at screening is positive for opioids, additional research-only screening activities will be performed. If applicable, a urine pregnancy test will be performed. If the urine pregnancy test is positive, the participant will not be eligible to continue, and the screening visit will be terminated. Participant demographic information will be collected followed by a psychiatric screening and assessment for alcohol and substance use disorder using the Mini-International Neuropsychiatric Interview (M.I.N.I. 7.0.2; 37) and for suicidality using the Columbia Suicide Severity Rating Scale (C-SSRS; 38). Participants will also undergo a physical examination and vitals assessment. Blood samples will be collected for complete blood count (CBC), comprehensive metabolic panel (CMP), and glycated hemoglobin (HbA1c) laboratory testing. Lastly, study coordinators will install the survey application (Wear-IT) on the participant’s phone; participants will receive instructions and practice completing the smartphone surveys of craving, stress, mood, pain, and sleep. If a participant does not have a smartphone, one will be loaned to the participant to be used during their study participation. To complete the screening for inclusion in the study, blood samples will be sent to a central clinical laboratory for assessment prior to randomization. The Screening Visit may be split over 2 days, if necessary to accommodate a participant’s schedule. If this is the case, the urine testing (drug screening and pregnancy testing), blood sample collection, and set up of smartphone surveys will take place on the 1st day of the visit. Randomization will occur after the Screening Visit (Study Week 1) and before the Baseline/Treatment Visit (Study Week 2), once the blood results have been returned and the participant is confirmed to be eligible to continue in the study. This will allow for study medication (active drug or placebo) to be available for administration at the Baseline/Treatment Visit. Participants will be randomized to either the semaglutide or placebo in a two-arm parallel group in a 1:1 ratio using a permuted-block randomization algorithm stratified by site and type of MOUD.

At the Baseline/Initial Treatment Visit (Study Week 2), a detailed medical history will be collected by the site physician and admission diagnoses will be confirmed. Participants will undergo vitals assessment, and a urine sample will be collected for the testing of substance use and to perform a pregnancy test (if of childbearing potential). Participants who become pregnant during the study will be asked if they are willing to complete the study visits and tasks as per the schedule, but without the administration of the study medication. The following psychiatric assessments will be conducted: C-SSRS for suicidal ideation, the Hamilton Depression Rating scale (HAM-D; 39, 40) for depressive symptoms, the State-Trait Anxiety Index (STAI; 41) for anxiety symptoms, the TLFB (42) for substance use history over the previous 30 days, an Opioid Craving Scale (OCS) to assess craving in the prior week, and the University of Rhode Island Change Assessment scale (URICA; 43) to assess motivation and readiness to change. Concomitant medications and adverse event information will be collected, and a Locator form will be completed to provide additional participant contact information. Collection of smartphone surveys will continue as described in the survey burst timing description. After all research activities are complete, the participant will receive a 0.25 mg dose of the study medication or placebo, depending on group randomization.

During the remaining treatment visits (Study Weeks 3–13), vitals, urine tests, and psychiatric assessments (C-SSRS, TLFB, and OCS) will be conducted at each visit; HAM-D and STAI will be conducted on Study Weeks 3, 5, 9, and 13. Collection of concomitant medications and adverse events (AEs) will continue at each visit and smartphone surveys will continue as described in the survey burst timing description. At each visit, following completion of the research activities, the participant will receive the study medication or placebo, depending on group randomization; participants will continue to receive a 0.25 mg dose on Study Weeks 3–5, a 0.5 mg dose on Study Weeks 6–9, and a 1.0 mg dose on Study Weeks 10–13.

At the washout visit (Study Week 14), vitals, urine tests, and psychiatric assessments (HAM-D, C-SSRS, TLFB, STAI, OCS) will be conducted and a blood sample will be collected for CBC, CMP, and HbA1c laboratory testing. Collection of concomitant medications and AEs will continue at each visit and smartphone surveys will continue as described in the survey burst timing.

The participant will have no research activities for Study Weeks 15–18, with the exception of smartphone survey completion in Study Week 18. At the follow-up visit (Study Week 19), vitals, urine samples for substance use testing, and final psychiatric assessments (C-SSRS, TLFB, and OCS) will be conducted. Concomitant medications and AEs will also be collected.

#### Financial Incentives

Participants will be offered a stipend for their time in the amount of $25 for completion of the screening visit (Study Week 1), $100 for completion of the Baseline/Treatment Visit (Study Week 2), $50 per visit for the remaining treatment visits (Study Weeks 3–13), $50 for the washout visit (Study Week 14), up to $98 for smartphone surveys and $50 for the follow-up visit (Study Week 19). An additional payment of $30 will be made at the follow-up visit for all participants who have used their own smartphone for the study, and for those participants who return the study phone they were issued. Participants issued with a study phone who do not return it at the follow-up visit will not receive the additional $30 payment. Payments will be titrated relative to the tasks accomplished during the study. Participants will earn $2 for each survey completed (defined as submission of 75% of answers in a given survey). The potential total amount of compensation is $903.00 (Table 2).

### Measures

#### Participant History

Demographic information (age, sex, race, contact information) will be collected on Study Week 1 at the screening visit. A detailed medical history and confirmation of admission diagnosis will be obtained on Study Week 2 (baseline visit). Concomitant medication information and AEs will also be collected throughout the study at their scheduled clinic visits (Study Weeks 2–14, 19).

#### Psychiatric Screening and Assessment

All participants will be administered the M.I.N.I. 7.0.2 (Modules I and J) and C-SSRS to assess alcohol/substance use disorders and suicidality, respectively. Any participant that reports recent suicidal ideation (past month) or behavior (past year) on the screening C-SSRS will be referred for further immediate evaluation by a clinician. Clinical judgment and additional consultation with the site PI will be used to further clarify if the participant is eligible to continue in the screening visit. On Study Weeks 3, 5, 9, and 13, the HAM-D (39, 40) will be administered to assess severity of depression and the STAI-Y1 (41) will be conducted to assess anxiety.

#### Physical Examination and Vitals Assessment

A physical examination will be conducted by the site physician on Study Week 1 (screening visit). Vitals assessments will be conducted at all study visits (screening, treatment, washout, and follow-up visits) and will include measurement of body weight, height (Week 1 only), heart rate, blood pressure, and respiratory rate. Height (inches) and body weight (lbs/kg) will be measured using standard height measurement tools and digital/analogue scales and will be used to calculate the participant’s BMI. Body weight will be monitored at the medication administration sessions and if the BMI falls below 18, but above 16, then it is up to clinical discretion for the participant to continue. If BMI falls below 16, the medication will be stopped, but the participant will remain in the study. Heart rate will be measured as beats per minute (bpm) using palpation of the radial artery. Blood pressure will be measured (mmHg) using a standard blood pressure monitor. Respiratory rate will be measured as breaths per minute via manual count.

#### Biospecimen Collection

All participants will provide a blood sample for laboratory testing (CBC, CMP, and HbA1c) at the screening and washout visits (Study Week 1, 14). If the blood sample is unable to be analyzed for any reason, the participant will be asked to return to have the blood samples re-drawn. Blood samples will be sent to a central clinical laboratory for analysis.

All participants will provide a urine sample at each study visit (Study Weeks 1–14, 19) to assess for the presence of opioids (including fentanyl), cocaine and other stimulants, benzodiazepines, barbiturates, and cannabis; and adherence to MOUD (BUP or methadone) and analyzed using point-of-care screens. If the urine sample is **not** positive for opioids at the screening visit, the participant will not continue in the study. For non-screening visits, if the participant reports no fentanyl use on the TLFB but the point-of-care urine sample is positive for fentanyl, the urine will be sent out to a commercial testing laboratory for quantitative analysis. If the participant is of childbearing potential, a urine pregnancy test will also be performed at weekly visits (Study Weeks 1–14) using point-of-care testing. Participants who become pregnant during the study will be asked if they are willing to complete the study visits and tasks as per the schedule, but without the administration of the study medication.

#### Assessments of Drug use and Craving

Smartphone surveys: Study coordinators will install the survey application (Wear-IT framework; 44) on the participant’s phone (or a phone loaned by the site) at the screening visit. The participants will receive instructions and practice completing the smartphone surveys of craving, stress, mood, pain, and sleep. Frequency and strength of craving will be assessed on 0-100-point Likert scales. Three items from the Desire for Drugs scale (adapted from the Desire to Drink scale; 45) will be assessed on 0–4 Likert scales to remain consistent with the prior literature. These items will include: “The idea of using drugs has intruded upon my thoughts”; “I have thought about how satisfying drugs can be”; and “I have missed the feeling drugs can give me.” Participants who do not have a compatible phone or who do not wish to install the app on their phone will be offered the loan of a phone from the study site. Participants will be encouraged to complete the survey at each timepoint and will be prompted regularly via the smartphone when a survey is available for completion. During the screening phase, participants will be prompted to answer daily surveys each day for seven days or until the first treatment visit, whichever is sooner. This data will serve as the baseline for participants who are then eligible to continue in the study. All data from participants who are not eligible to continue the study will be deleted from the data set. Participants will then complete daily surveys in bursts of 7 days/week periodically throughout the treatment intervention (Study Weeks 2, 5, 9, 13), for the washout period (Study Week 14), and for a week during the follow-up period (Study Week 18).

The URICA will be administered at the baseline visit (Study Week 2) to assess motivation and readiness to change drug use. A modified TLFB and an OCS also will be administered at study visits (Study Weeks 2–14, 19) to obtain measures of polydrug use and craving/motivation, respectively. The TLFB is a clinical research tool designed to obtain quantitative estimates of cannabis, cigarette, and other drug use (42). A modified TLFB was created for use in the current protocol and will be administered by a trained interviewer to collect recent days of substance use for opioid analgesics, heroin, fentanyl, alcohol, nicotine, cannabinoids, cocaine, crack, amphetamine-type stimulants, hallucinogens, sedatives and hypnotics, benzodiazepines, and inhalants. The OCS will consist of five craving questions and two motivation questions. The five craving questions will be those presented in the smartphone surveys including frequency and strength of craving and the 3 items from the Desire for Drugs scale. The motivation questions will be from taken from the URICA and will be “Even though I’m not always successful in changing, I am at least working on my problem” and “I may need a boost right now to help me maintain the changes I’ve already made”. Participants will answer these questions retroactively (at the study visit), unlike the momentary response collected in the smartphone surveys.

#### Study Treatment Dose Regimen

The recommended maximum dosage is 1 mg/week, administered at any time of day. This choice in dosage is based on the current FDA-approved dosing regimen for semaglutide. Semaglutide will be administered on a fixed flexible schedule, and while there will be set points to increase the dose, we will rely on clinician judgment if a participant would like to stay at or return to a lower dose. The planned dosing schedule will start with the initial dose at 0.25 mg once weekly for 4 weeks. The dosage will then be increased to 0.50 mg once weekly for 4 weeks, and finally, will be increased to 1.0 mg once weekly for 4 weeks. If the participant does not wish to increase their dose (due to, for example, GI distress), they may, with clinician judgment and approval, remain at a lower dose for the subsequent weeks. Participants may choose to stop their study medication at any point during the treatment period and then restart. The dose with which the participant restarts is at the discretion of the site PI.

Participants will be allowed to switch MOUD (e.g., from BUP to methadone) during the study. They will continue their current dose of the study medication (active drug or placebo) at the time of the switch and will not have to start again at the lowest dose. Participants who choose to discontinue their MOUD, but who wish to remain on the study medication (active drug or placebo) will be allowed to continue in the study and receive study medication.

### Data Management and Monitoring

#### Early Withdrawal of Subjects and Safety Reporting

Participants will be withdrawn from the study if the participant withdraws consent for any reason, engages in behavior that could jeopardize their own health and well-being or that of others, or for loss of capacity to consent (e.g., overdose, severe stroke, coma, dementia). An attempt to locate a legally authorized representative (LAR) will be made for participants withdrawn due to loss of capacity to request LAR consent for up to 5 months of SAE follow up. If the study team becomes aware that a participant has become incarcerated (a prisoner) during the study and the participant will not be released prior to what would have been the end date of their participation in the study, the participant will be withdrawn from the study. If the participant will be released prior to the end of their participation in the study, the study team will suspend research procedures and interactions with the participant during their incarceration. Any smartphone data collected while the participant was a prisoner will be deleted.

Participants who express a desire to withdraw, are withdrawn from the study medication or become pregnant during the study, will be asked if they are willing to continue the study, but without the study medication. If they agree to continue in the study, they will complete the study visits and tasks as per the schedule, but without the administration of the study medication (intention-to-treat). Participants who become pregnant during the study will be asked to complete an additional consent form allowing the study team to follow up on the outcome of their pregnancy.

The study PI and medical monitor will review data and safety in real-time throughout the study. Participants will be routinely questioned about AEs at study visits. All AEs (serious or non-serious) and abnormal test findings observed or reported to the study team will be followed until the event (or its sequelae) or the abnormal test finding resolves or stabilizes at a level acceptable to the investigator. The study PI will confirm that all AEs are correctly entered into the AE case report forms in REDCap by the coordinator, be available to answer any questions that the coordinators may have concerning AEs, and will notify the IRB and/or Data and Safety Monitoring Board (DSMB) of all applicable AEs, as appropriate. All assessments of AEs will be made by a licensed medical professional who is an investigator on the research study. In the event of a report of a serious AE (SAE) or life-threatening adverse reaction, the study site staff will inform the study PI within 24 hours of obtaining knowledge of the event.

#### Data and Safety Monitoring Plan

The DSMB will provide an objective review of treatment results as they relate to participant safety and data quality. The sponsor-approved DSMB will review study progress, safety of study participants, and progress of enrollment every 6 months.

The DSMB will be responsible for safeguarding the interests of participants in this trial. This responsibility will be exercised by providing recommendations for continuation or early termination of the trial, based on assessment of safety. The DSMB may also formulate recommendations related to the selection, recruitment, or retention of participants, their management and adherence to protocol-specified interventions, and procedures for data management and quality control. The DSMB will be advisory to the study PI, to the medical monitor, and to the co-investigators. The study PI will be responsible for promptly reviewing and implementing DSMB recommendations.

DSMB membership will be restricted to individuals who have no apparent financial, scientific, or regulatory conflicts of interest. DSMB members are expected to declare any other potential conflicts of interest so that other members can judge whether any such conflicts might affect objectivity. Members who develop significant conflicts of interest during the course of the trial are expected to resign from the DSMB. Members of the DSMB are otherwise expected to continue their participation until the trial is closed. If necessary, the study PI will appoint replacements for any DSMB members who resign.

#### Clinical Trial Monitoring Plan

The study will be monitored by the Clinical Trial Monitoring Team at the Penn State College of Medicine. Staff conducting the study will have up-to-date Good Clinical Practice (GCP) and Human Research Subjects Protection (HRSP) trainings, and any additional research-related trainings as required by the site institutions. Prior to the first pre-screening (review of EMR) at each site, study staff will undergo training on the protocol and the data collection systems. The Clinical Trial Monitoring Team will monitor the study for protocol compliance, data quality, and regulatory compliance. This will include review of the informed consent process and completed forms, verification of the presence of essential documents in the study regulatory binder, completion of source document verification for data entered into Research Electronic Data Capture (REDCap), ensuring that the study is implemented as planned, review of AEs and the reporting of SAEs, and ensuring that all data quality rules have been executed and resolved and all data queries are resolved and closed.

#### Statistical analysis

The primary outcome is the probability of abstinence from illicit, non-prescribed opioids, a binary repeated measure over the 12 treatment weeks of the trial. A power analysis using G*Power indicated that our proposed sample size of N = 100 per group will provide 80% power to detect a proportional difference of relatively small size (odds ratio 1.7) between the treatment and control groups. This corresponds to abstinence in an additional 15–20% of the participants in the control group.

Primary outcome analysis: A mixed-effects generalized linear model (GLM) with a logit link will be fit, modeling log-odds of abstinence as a function of treatment assignment (semaglutide vs placebo), time (treatment weeks), and covariates MOUD type (methadone; BUP), and site (PPI; UMD; NYU). The main effect of treatment assignment, i.e., the coefficient of the treatment term and its associated odds ratio and confidence limits, will be the test of treatment effect. Interactions between treatment assignment and covariates will be fit and retained in the model if significant.

Secondary outcomes analysis: Smartphone surveys data yields continuous and Likert-type scores over multiple repeated measurements. Drug craving scores derived from the smartphone surveys will be analyzed as a mixed-effects GLM. Specifically, linear mixed-effects GLMs will be used to evaluate the difference in the level and trend over time of self-reported craving between treatment groups. Baseline craving scores will be included as covariates, along with MOUD type (methadone; BUP) and site. Participants with intermediate missing values will be incorporated in the mixed-effects analysis under the assumption that data are missing at random. Analogous models will be fit for other repeated continuous or count measures such as in-person Cravings Scales, or days using substances from TLFB. Sensitivity analyses will examine alternative assumptions about missingness, including missing at random.

For discrete binary outcomes (e.g., abstinent over last 4 weeks of the trial; retained on MOUD at treatment week 12) logistic regression will be used to model outcome as a function of treatment assignment, MOUD type, and site.

SAS (version 9.4) and R (version 4.3.2 or latest current version) software will be used for analyses with a significance level of 0.05. Summary statistics including means, medians, ranges, and standard deviations will be computed for each continuous variable, as well as distributional assumptions, and frequencies computed with percentages for categorical variables. Identified data outliers will be further examined to determine if they are data entry errors, in which case they will be modified when possible; otherwise, values will be kept for final analysis.

## Discussion

This manuscript describes the protocol for a randomized, double-blind, placebo-controlled, clinical trial designed to evaluate the efficacy of the GLP-1RA, semaglutide, as a treatment for individuals with MOUD-refractory OUD in an outpatient setting. The proposed study builds upon our previous data demonstrating a reduction in craving as measured by EMA with the GLP-1RA, liraglutide, and extends the use of GLP-1RAs from a residential to outpatient population. We plan to assess the probability that participants will be abstinent from illicit and nonprescribed opioids at the end of a 12-week treatment with semaglutide or placebo as determined by urine testing and self-report with the TLFB. Secondarily, we will also assess opioid craving using EMA, craving and its association with abstinence from opioids and other drugs, and the days of opioid and stimulant use across the 12-week treatment.

### Key Strengths and Limitations

A strength of the current proposal is that it extends evaluation of GLP-1RAs from a residential treatment population into an outpatient population that is engaged in treatment with MOUD. Residential treatment plays an important role in the treatment plan of OUD recovery; however, it is often comparatively short-term with an average stay of 30 days (46). The timeframe for GLP-1RA effects after discontinuation remains to be fully evaluated, but current evidence suggests that GLP-1RAs are effective while present in the body (47). It is therefore important that patients continue to receive support following a residential program and the use of MOUD provides that support; it provides a long-term maintenance program and allows the patient to reintegrate into their personal environment and daily lives while continuing treatment. As such, the potential to utilize GLP-1RAs in conjunction with MOUD is an important avenue of research to pursue that will be addressed in the current design.

Relatedly, the emphasis on treatment-refractory patients within populations utilizing MOUD has both strengths and limitations. In evaluating this subgroup of MOUD patients, the study examines an alternative treatment option for patients with relatively few options when standard MOUD treatments are ineffective. This is an avenue of research with significant therapeutic need and potential; however, it is also important to note that this population is inherently difficult to treat. Consequently, the therapeutic effect size of GLP-1RAs in this subpopulation may be smaller relative to the overall MOUD population. This assumption, however, is an empirical question that the current study aims to investigate.

Another important factor to consider is the sample size of the current study, which proposes to include 200 participants. This number is significantly larger than that of our previous pilot study, which had only 20 participants, and will be conducted across multiple sites, which will allow for a more diverse population then previously evaluated. However, while the proposed sample size is adequate to address the research aims outlined in this manuscript, it is not sufficient to support a new indication for GLP-1RAs as a treatment for OUD. Therefore, in addition to the current protocol, a larger phase III clinical trial will be needed to support a new indication. This study serves as a preliminary step towards that goal.

A limitation of the current protocol is that it does not address the stand-alone efficacy of a GLP-1RA compared with its efficacy as an adjunct to MOUD. Many people will not use currently available MOUD due to familial or work-related stigma, and several professions do not allow the use of full or partial opioid agonists while on the job (e.g., physicians, pilots). New pharmacological options are needed that circumvent these treatment barriers. GLP-1RAs may have the potential to fulfill this role if future studies determine that they can provide sufficient efficacy to prevent return to use among individuals in OUD treatment.

### Anticipated Impact

Preclinical models suggest that the use of a GLP-1RA significantly reduces heroin and fentanyl seeking and taking in rats and our prior clinical data suggest that a GLP-1RA can reduce opioid craving in humans. However, no GLP-1RA is currently approved to treat OUD in humans. Completion of the current protocol could provide additional support for the use of GLP-1RAs as a medication for OUD and allow physicians to include GLP-1RAs as an adjunct treatment option with current MOUD programs. Such an option would have significant potential for those with treatment refractory OUD and would improve lives by expanding the number of diverse, safe, and effective treatments for OUD.

## Figures and Tables

**Table 1:** Schedule of Study Activities

	Screening	Randomization	Treatment Intervention (all weeks have a + 5-day window, with the exception of V1, which has a + 14-day window)												Washout (+5-day window)	Follow-up (+1-month window)
Visit	0		1	2	3	4	5	6	7	8	9	10	11	12	13	14
**Study Week**	1		2	3	4	5	6	7	8	9	10	11	12	13	14	19
Study medication (active drug or placebo)			**0.25 mg active drug or placebo**				**0.50 mg active drug or placebo**				**1.0 mg active drug or placebo**					
			X	X	X	X	X	X	X	X	X	X	X	X		
Informed consent	X															
Urine sample for drug screening	X		X	X	X	X	X	X	X	X	X	X	X	X	X	X
Review inclusion/exclusion criteria	X															
MINI	X															
C-SSRS	X		X	X	X	X	X	X	X	X	X	X	X	X	X	X
Urine pregnancy test*	X		X	X	X	X	X	X	X	X	X	X	X	X	X	
Physical examination	X															
Vitals (Height**, weight, HR, BP, RR)	X		X	X	X	X	X	X	X	X	X	X	X	X	X	X
Blood sample (CBC, CMP, HbA1c)	X														X	
Collect demographics	X															
Medical history, including admission diagnoses			X													
Con meds			X	X	X	X	X	X	X	X	X	X	X	X	X	X
HAM-D			X	X		X				X				X	X	
STAI			X	X		X				X				X	X	
TFLB			X	X	X	X	X	X	X	X	X	X	X	X	X	X
URICA			X													
In-person cravings scales			X	X	X	X	X	X	X	X	X	X	X	X	X	X
Randomize		X														
Smartphone surveys setup, training	X															
Smartphone surveys	X		X			X				X				X	X	X***
AEs and SAEs	X		X	X	X	X	X	X	X	X	X	X	X	X	X	X
Return equipment																X

* If the participant has not notified the study team of current pregnancy;

** Height only collected at Enrollment/Baseline visit;

*** Only during study week 18, four weeks after study week 14

**Table 2:** Potential compensation amounts for each treatment arm

	Financial Incentives	Maximum Incentive
Screening Visit	$25	$25
Baseline Visit/Treatment Visit 1	$100	$100
Treatment Visits 2–12	$50/visit	$550
Washout Visit	$50	$50
Follow Up Visit	$50	$50
Returned Equipment/Use of own Phone	$30	$30
Smart Phone Survey Completion	$2/survey	$98
**Total Compensation**		**$903**

## Data Availability

Upon reasonable request, which should be made to the corresponding author, study data or materials may be made available.

## Abbreviations

AE: adverse event
AUD: alcohol use disorder
BMI: body mass index
BUP: buprenorphine
CBC: complete blood count
CDC: Centers for Disease Control and Prevention
CMP: comprehensive metabolic panel
C-SSRS: Columbia Suicide Severity Rating Scale
DPP-4: dipeptidyl peptidase-4
DSMB: data and safety monitoring board
EMA: ecological momentary assessment
EMR: electronic medical record
FDA: Food and Drug Administration
fMRI: functional magnetic resonance imaging
GCP: Good Clinical Practice
GFR: glomerular filtration rate
GI: gastro-intestinal
GLM: generalized linear model
GLP-1RA: glucagon-like peptide 1 receptor agonist
HAM-D: Hamilton Depression Rating scale
HbA1c: glycated hemoglobin
HRSP: Human Research Subjects Protection
iOAT: injectable opioid agonist treatment
IRB: Institutional Review Board
MEN 2: multiple endocrine neoplasia syndrome Type 2
MINI: Mini-International Neuropsychiatric Interview
MOUD: medication for opioid use disorder
MTC: medullary thyroid carcinoma
NST: nucleus of the solitary tract
NYU: New York University at Bellevue Hospital Center
OCS: Opioid Craving Scale
OUD: opioid use disorder
OTP: opioid treatment program
PI: Principal Investigator
PPI: Pennsylvania Psychiatric Institute
RCC: recovery community center
REDCap: research electronic data capture
SAE: serious adverse event
SROM: slow-release oral morphine
STAI: State-Trait Anxiety Index
SUD: substance use disorder
TLFB: Timeline Followback
UMD: University of Maryland Baltimore
